# Factors influencing the fascial closure rate after open abdomen treatment: Results from the European Hernia Society (EuraHS) Registry: Surgical technique matters

**DOI:** 10.1007/s10029-020-02336-x

**Published:** 2020-11-21

**Authors:** A. G. Willms, R. Schwab, M. W. von Websky, F. Berrevoet, D. Tartaglia, K. Sörelius, R. H. Fortelny, M. Björck, T. Monchal, F. Brennfleck, D. Bulian, C. Beltzer, C. T. Germer, J. F. Lock, EURAHS Open Abdomen Group, C. Güsgen, S. Schaaf, F. Anger, S. Fuhr, M. Kiesel, R. Schmidt, J. C. Kalff, T. O. Vilz, C. Galatioto, L. Cobuccio, A. Hoffmann, H. J. Schlitt, M. Heiss, F. Muysoms, K. Oldhafer, U. Dietz, Martin Björck, A. Vanlander

**Affiliations:** 1 Department of General, Visceral and Thoracic Surgery, German Armed Forces Central Hospital of Koblenz, Rübenacher Str. 170, 56072 Koblenz, Germany; 2 Department of General, Visceral, Thoracic and Vascular Surgery, University Hospital of Bonn, Sigmund-Freud-Str. 25, 53127 Bonn, Germany; 3 Department of General and HPB Surgery and Liver Transplantation, Ghent University Hospital, Corneel Heymanslaan 10, 9000 Ghent, Belgium; 4 Emergency Surgery Unit, Cisanello University Hospital, Via Paradisa 1, 56124 Pisa, Italy; 5 Department of Surgical Sciences, Section of Vascular Surgery, Uppsala University, SE 751 85 Uppsala, Sweden; 6 Department of Vascular Surgery, Rigshospitalet, University of Copenhagen, Blegdamsvej 9, 2100 Copenhagen, Denmark; 7 Faculty of Health and Medical Sciences, University of Copenhagen, Blegdamsvej 3B, 2200 Copenhagen, Denmark; 8 Department of General, Visceral and Oncological Surgery, Wilhelminenspital, 1160 Vienna, Austria; 9 Medical Faculty, Sigmund Freud University of Vienna, 1020 Vienna, Austria; 10 Department of General Surgery, Sainte Anne Military Hospital, 2 Boulevard Sainte-Anne, 83000 Toulon, France; 11 Department of Surgery, Regensburg University Hospital, Franz-Josef-Strauß-Allee 11, 93053 Regensburg, Germany; 12 Department of Abdominal, Tumor, Transplant and Vascular Surgery, Cologne-Merheim Medical Center, Witten/Herdecke University, Ostmerheimer Str. 200, 51109 Cologne, Germany; 13 Department of General, Visceral and Thoracic Surgery, German Armed Forces Hospital of Ulm, Oberer Eselsberg, Ulm, Germany; 14 Department of General, Visceral, Transplantation, Vascular and Pediatric Surgery, University Hospital of Würzburg, Oberdürrbacher Str. 6, 97080 Würzburg, Germany

**Keywords:** Open abdomen, Peritonitis, Fascial closure, Hernia, Abdominal compartment syndrome, Abdominal trauma, Burst abdomen, NPWT, VAC

## Abstract

**Purpose:**

Definitive fascial closure is an essential treatment objective after open abdomen treatment and mitigates morbidity and mortality. There is a paucity of evidence on factors that promote or prevent definitive fascial closure.

**Methods:**

A multi-center multivariable analysis of data from the Open Abdomen Route of the European Hernia Society included all cases between 1 May 2015 and 31 December 2019. Different treatment elements, i.e. the use of a visceral protective layer, negative-pressure wound therapy and dynamic closure techniques, as well as patient characteristics were included in the multivariable analysis. The study was registered in the International Clinical Trials Registry Platform via the German Registry for Clinical Trials (DRK00021719).

**Results:**

Data were included from 630 patients from eleven surgical departments in six European countries. Indications for OAT were peritonitis (46%), abdominal compartment syndrome (20.5%), burst abdomen (11.3%), abdominal trauma (9%), and other conditions (13.2%). The overall definitive fascial closure rate was 57.5% in the intention-to-treat analysis and 71% in the per-protocol analysis. The multivariable analysis showed a positive correlation of negative-pressure wound therapy (odds ratio: 2.496, *p* < 0.001) and dynamic closure techniques (odds ratio: 2.687, *p* < 0.001) with fascial closure and a negative correlation of intra-abdominal contamination (odds ratio: 0.630, *p* = 0.029) and the number of surgical procedures before OAT (odds ratio: 0.740, *p* = 0.005) with DFC.

**Conclusion:**

The clinical course and prognosis of open abdomen treatment can significantly be improved by the use of treatment elements such as negative-pressure wound therapy and dynamic closure techniques, which are associated with definitive fascial closure.

**Electronic supplementary material:**

The online version of this article (10.1007/s10029-020-02336-x) contains supplementary material, which is available to authorized users.

## Introduction

Open abdomen treatment (OAT) involves the deliberate decision not to close the fascia at the end of laparotomy [1, 2]. This surgical strategy is used in the management of critically ill patients with serious intra-abdominal conditions [3–5], e.g. severe secondary peritonitis, abdominal trauma, or abdominal compartment syndrome.

Since its introduction, OAT has become an established treatment strategy [1, 6, 7]. Its objectives are to reduce the extent of initial surgery as part of a damage control or abbreviated laparotomy strategy, to temporarily close the abdomen in a sterile environment and tension-free environment, and to facilitate second-look operations. Morbidity and mortality rates can thus be reduced in critically ill patients whose systemic compensatory mechanisms are depleted and whose physiological reserves are nearly exhausted [3–6, 8].

OAT serves as a preventive and therapeutic measure in the septic abdomen, in damage control situations and when the abdominal compartment syndrome (ACS) has developed. The main objective once the abdominal situation is under control is to achieve definitive fascial closure (DFC) (i.e. definitive fascial closure) as soon as possible to decrease secondary morbidity since early DFC is associated with lower mortality and complication rates [9–11].

Treatment without fascial closure has severe consequences not only during but also after a hospital stay. Giant planned ventral hernias are an inevitable result and require complex secondary reconstructive procedures, which are also associated with considerable risks [12–14]. Quality of life, which is an important parameter for assessing the condition of patients after a hospital stay, is severely reduced in patients with giant ventral hernias [15, 16].

The combination of different OAT elements such as vacuum-assisted wound closure and mesh-mediated fascial traction (VAWCM), negative-pressure wound therapy (NPWT) and dynamic fascial sutures (DFS) is associated with the highest frequency of DFC [11, 16–19]. The purpose is to create synergistic effects of edema reduction and gradual fascial approximation [18, 20–22].

OAT with VAWCM represents the current gold standard with fascial closure rates of up to 90% and is acknowledged to be superior to other techniques without mechanical fascial traction, which provide maximum fascial closure rates of up to 60% [3, 9, 21, 23–26]. For this reason, the current European Hernia Society (EHS) guidelines already recommend the use of dynamic closure techniques (DCTs) but point out that the level of evidence is low [27].

Research work has not yet systematically addressed and analyzed the role of different potentially crucial patient and surgical factors. The focus of previous studies has so far been on comparing different techniques that comprise several different factors. A further question is whether the various treatment factors play the same role regardless of the underlying indication for OAT.

We therefore chose this multi-center registry approach to investigate what patient-related and which surgical technical factors have an impact on DFC in OAT.

## Material and methods

### Registry

Since 1 May 2015, every hospital can enter data online into the Open Abdomen Route, which is a registry of the European Hernia Society (EHS) that was established as a module of the European Registry of Abdominal Wall Hernias (EuraHS – www.eurahs.eu). In cooperation with the EHS, the registry was implemented by a working group of surgeons from the Department of General, Visceral, Transplantation, Vascular and Pediatric Surgery at the University Hospital of Würzburg and the Department of General, Visceral and Thoracic Surgery at the Federal Armed Forces Central Hospital in Koblenz. The EuraHS IT platform was developed by the Department of Artificial Intelligence and Applied Informatics of the Institute for Mathematics and Computer Science at the University of Würzburg in Germany [28]. EuraHS was established in 2012 and currently provides six different registries (routes). The Open Abdomen Route was created to allow data to be collected and analyzed from open abdomen patients from multiple centers in a systematic and standardized manner and thus to achieve a higher level of evidence [29].

The Open Abdomen Route covers 110 variables on every patient and course of treatment and is divided into 11 categories that provide information on the treating hospital, the patient, underlying conditions and comorbidities, open abdomen management, clinical course, and three clinical follow-up assessments (Supplement 1).

### Data collection

All data are pseudonymized so that only the responsible hospital can re-identify patients. A case-related identification number is assigned to every patient during registration.

The study was approved by the ethics commission of the State of Rhineland-Palatinate, Germany [No.: 837 534 13 (9219-F) as well as in the ethical review boards of the hospitals entering data. Data entry requires previous written informed consent from the patient or a legal representative. Additionally, the study was registered in the International Clinical Trials Registry Platform via the German Registry for Clinical Trials (DRK00021719). The EuraHS data protection regulations and publication guidelines must be observed [28]. The Open Abdomen Route is supported by an advisory panel from CAMIN (Surgical Working Group for Military and Emergency Surgery), which is a working group of the DGAV (German Society for General and Visceral Surgery). For the present analysis, a separate Open Abdomen Study Group was formed within the Open Abdomen Route. All members gave their consent for participation and the anonymous use of data by the initiators of the registry.

### Patient inclusion

#### Study period

All cases entered into the registry between 1 May 2015 and 31 December 2019 were included in the present analysis.

#### Participating hospitals

The patients whose data were analyzed here underwent treatment at hospitals that are members of the Open Abdomen Study Group, which was formed within the Open Abdomen Route (www.eurahs.eu). Data were provided by eleven hospitals (Supplement 2).

#### Inclusion criteria

• All OAT patients.

• All indications and underlying diseases.

• All types of OAT-related surgical techniques.

• Minimum survival > 24 h after initiation of OAT (index procedure).

• Informed consent (patient or legal representative).

Exclusion criteria

• No informed consent.

• Death within 24 h after the initiation of open abdomen management.

• Data set for the variables included in the analysis was incomplete.

• Death before termination of OAT led to exclusion from the analysis of DFC (endpoint) (per-protocol analysis).

### Definitions

*Study endpoint*: definitive fascial closure (DFC) after completion of OAT in a per-protocol analysis.

According to the EHS, DFC is any situation where the fascia is completely closed, i.e. the fascial edges are completely sutured together with no remaining fascial defect (fascia-to-fascia closure) [27]. DFC was not achieved if a fascial gap was present, regardless of whether alloplastic mesh had been used to bridge the defect or augment or replace the fascia.

Patients who died before the completion of OAT were excluded from the multivariate analysis of DFC.

The group of patients in which the study endpoint DFC was not achieved thus comprised all cases in which abdominal closure had been achieved using one of the following techniques:

- Closure of skin and subcutaneous tissue only (planned ventral hernia)
- Granulation of the abdominal wall defect and subsequent coverage of the defect with a split-thickness skin graft
- Granulation tissue formation through Vicryl® (polyglactin 910) mesh, which was placed in inlay position to bridge the defect, and subsequent coverage with a split-thickness skin graft
- All uses of alloplastic mesh (intraperitoneal onlay mesh, inlay, onlay, sublay) in which a fascial defect was bridged and complete fascial closure was not achieved

#### Open abdomen treatment (OAT) elements

The different types of open abdomen treatment elements and techniques were investigated on the basis of three aspects:

- Management of the fascia
- Management of the viscera
- Application of negative-pressure wound therapy

#### Dynamic closure techniques (DCT)

DCT comprises all techniques that use dynamic procedures to gradually and actively reduce the fascial defect. Used techniques were mesh-mediated fascial traction and dynamic fascial sutures. Simple mesh interposition without gradual reduction of the fascial defect was not considered a DCT.

#### Visceral protective layer (VPL)

VPL includes all techniques that involve the intra-abdominal placement of an inert non-adhesive protective layer as deep as possible into the paracolic gutters, the retropubic space and the subxiphoidal region as this was shown to reduce enteroatmospheric fistula (EAF) formation [30].

#### Negative-pressure wound therapy (NPWT)

NPWT includes all techniques in which negative pressure or suction is actively applied to remove exudate. Drains (Jackson–Pratt drains, Robinson drains, etc.) that are used for the passive removal of fluids do not meet this requirement.

#### Classification of the open abdomen

The study is based on Björck’s initial classification that was published in 2009 and amended in 2016 [31, 32]. This system identifies four grades of the open abdomen.

#### Sepsis

Until 2016 we used the definitions for sepsis as proposed by Bone et al. in 1992. Since 2016, we have used the Third International Consensus definitions as published by Singer et al. [33, 34].

#### Abdominal compartment syndrome (ACS)

ACS was defined as a sustained intra-abdominal pressure (IAP) increase above 20 mmHg with at least one new-onset organ dysfunction or, alternatively, an abdominal perfusion pressure (APP) above 60 mmHg with at least one new-onset organ failure [35].

### Statistical analysis

Data was analyzed using descriptive statistical methods. Metric variables were tested for normal distribution using the Kolmogorov–Smirnov test. Normally distributed variables were presented as means ± standard deviations. Non-normally distributed variables were presented as medians and 95% confidence intervals (CIs). Depending on the distribution and the level of measurement, univariate analyses were performed using Fisher’s exact test, the chi-squared test or the Mann–Whitney *U* test. The significance threshold was set at *p* = 0.05.

We performed a multivariate logistic regression analysis to identify possible factors that influence the target variable. DFC yes/no was defined as the dependent variable (target variable) and potential influencing factors were defined as independent variables. The constellation of variables in the regression model was formed by means of the inclusion method, i.e. all selected independent variables were included, and a forward stepwise analysis was performed (likelihood ratio). Furthermore, a subgroup analysis for the two largest subgroups “peritonitis” and “ACS” was performed to address the question of whether the various treatment factors play the same role in different OAT indications.

We calculated the coefficient of determination (Nagelkerke’s pseudo *R*^2^), the odds ratio to evaluate the regression model and effect size.

Data were processed and analyzed using Excel (2010, Microsoft, Redmond, United States), XLSTAT (Addinsoft, Paris, France), and SPSS (version 25, IBM, Armonk, United States).

## Results

From 1 May 2015 to 31 December 2019, 679 data sets that had been provided by the Open Abdomen Study Group were retrieved from the Open Abdomen Route via www.eurahs.eu. Of these, 630 data sets offered complete information on the variables investigated in this study and were provided by eleven hospitals which provide tertiary or advanced secondary care in six European countries.

**Fig. 1:**
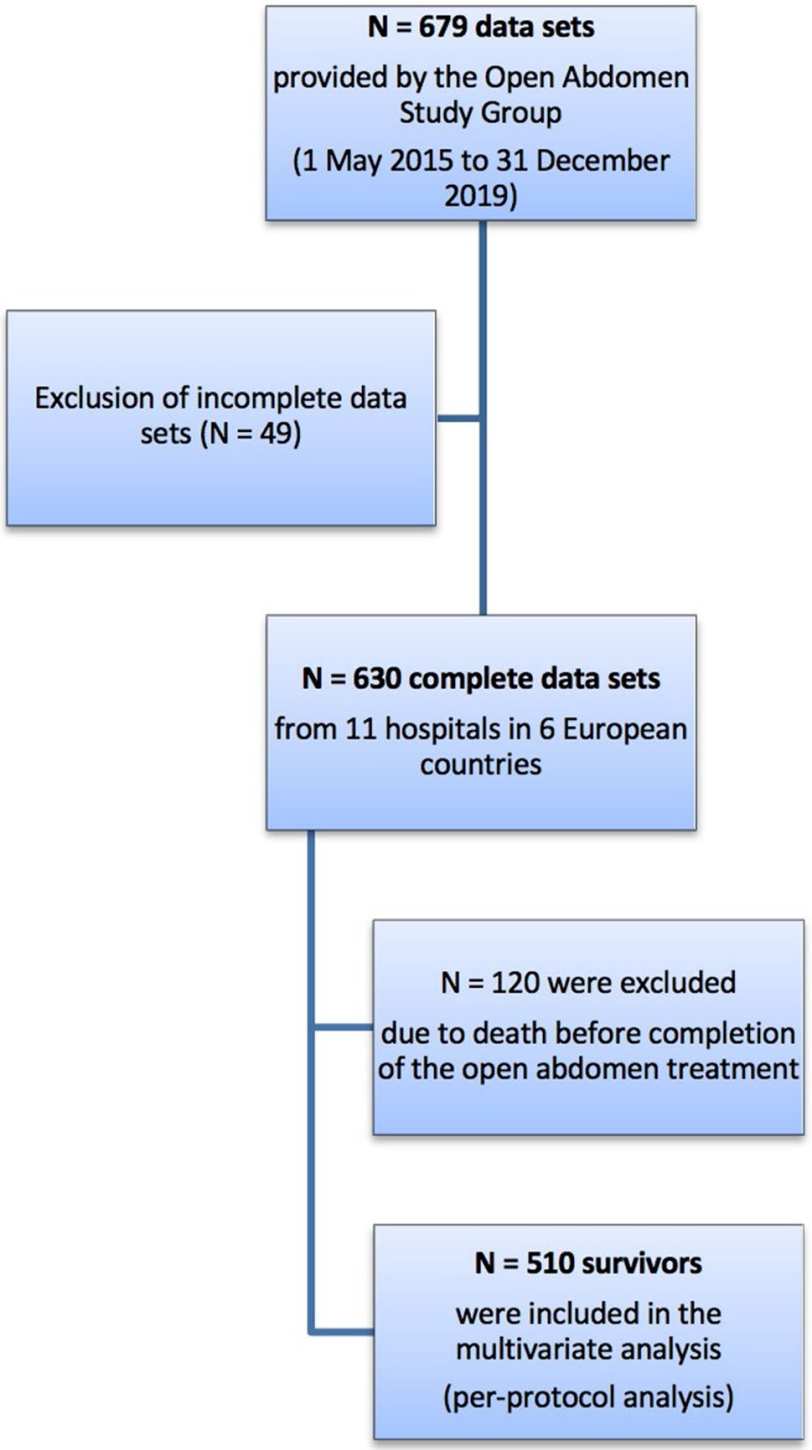
Prism flow chart of patient inclusion

In accordance with the study protocol, patients who died before the completion of OAT were excluded from the multivariable analysis, which examined the effects of a variety of factors on DFC. The analysis thus included 510 survivors in the per-protocol analysis (Fig. 1).

### Demographic data

The mean age of the patients was 59.8 ± 15.9 years. Two thirds of them were male. The mean body mass index (BMI) was 28.7 at the initiation of OAT. Table 1 provides an overview of data and comorbidities of the patients included in this study.

**Table 1:** Patient and treatment characteristics

**Patient characteristics**	
Number of patients	630
Age (years)	59.82 ± 15.9 (median: 61.62)
Gender (female/male)	210 (33.3%)/420 (66.7%)
Body mass index (BMI)	28.7 ± 23.9 (median: 26.12)
Malignancy	141 (22.4%)
Diabetes	91 (14.4%)
Cardiopulmonary disease	253 (40.2%)
Immunosuppression	52 (8.3%)
Mannheim Peritonitis Index (MPI)	21 ± 8 (median: 22)
Injury Severity Score (ISS)	25 ± 20 (median: 26)
**Treatment characteristics**	
Mortality	120/630 (19%)
Fistula incidence	9%
Length of stay before OAT (days)	9.05 ± 15.26 (median: 4)
OAT after first operation yes/no	305 (48.4%)/325 (51.6%)
Surgical procedures before OAT	1 (*n* = 202/32%)
	2 (*n* = 81/12.9%)
	3 (*n* = 29/4.6%)
	4 (*n* = 13/2.1%)
Type of incision (midline/transverse/combined)	502 (79.7%)/56 (8.9%)/12 (1.9%)
Indication for previous surgery (elective/emergency)	198 (31.4%)/419 (66.5%)
Intra-abdominal contamination at the initiation of OAT (yes/no)	290 (46%)/340 (54%)
Sepsis at the initiation of OAT (yes/no)	228 (36.2%)/402 (63.8%)
Björck’s classification at the initiation of OAT	Grade 1A—clean OA (27.9%)
	Grade 1B—contaminated OA (21.4%)
	Grade 2A—clean OA developing adherence (12.0%)
	Grade 2B—contaminated OA developing adherence (33.1%)
	Grade 3—OA complicated by fistula (2.6%)
	Grade 4—frozen OA (2.9%)
Björck’s classification at the completion of OAT	Grade 1A—clean OA (26.5%)
	Grade 1B—contaminated OA (1.5%)
	Grade 2A—clean OA with adherence (51.4%)
	Grade 2B—contaminated OA with adherence (2.9%)
	Grade 3—fistula (9.7%)
	Grade 4—frozen abdomen (5.8%)

*OAT* open abdomen treatment, *OA* open abdomen

Overall hospital mortality was 19% (120 of 630 patients). Table 1 provides an overview of the epidemiology, comorbidities and outcome of the patients included in this study.

### Indications for the open abdomen

**Fig. 2:**
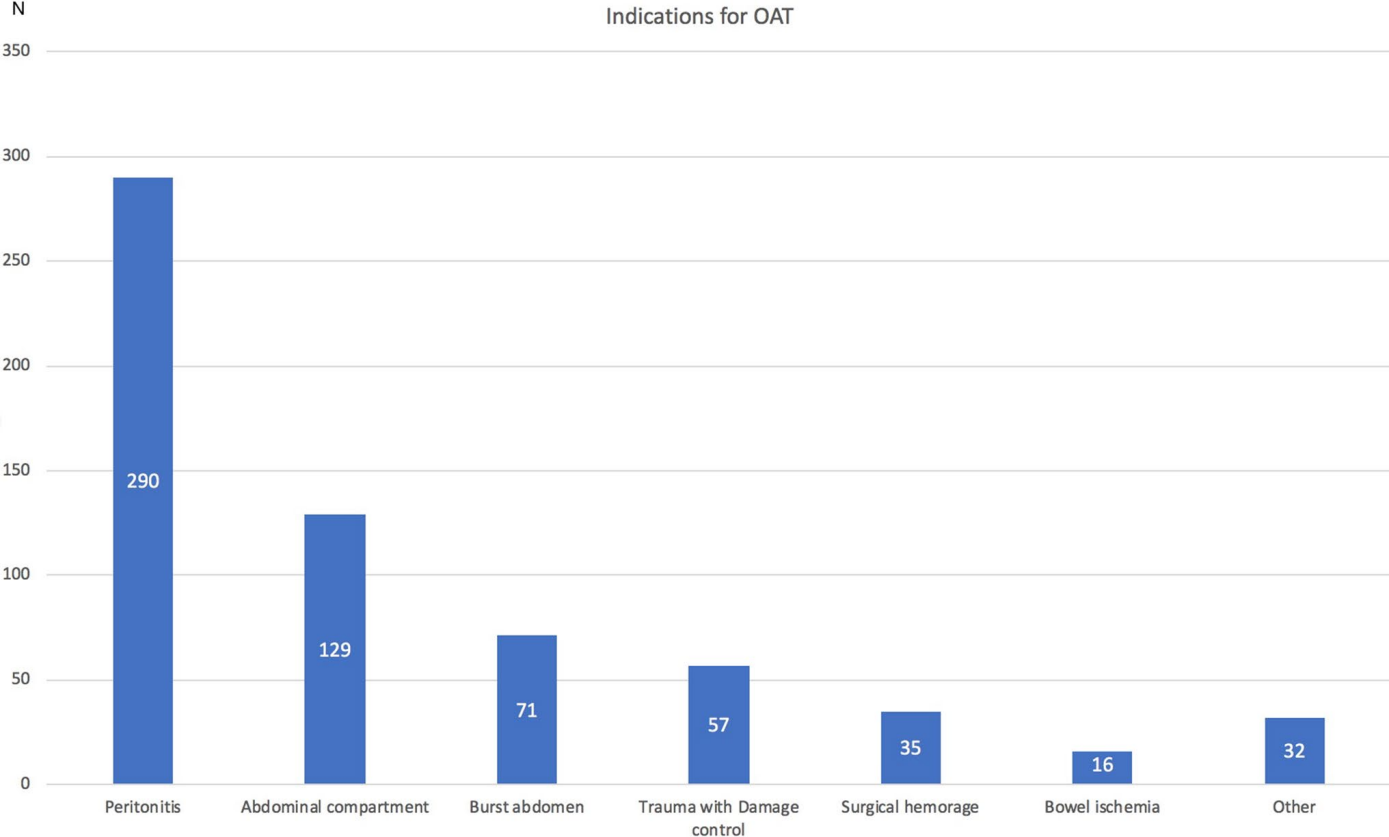
Indications for the open abdomen (number of patients)

The majority of patients required OAT for peritonitis (46%), followed by abdominal compartment syndrome (ACS) (20.5%), burst abdomen (11.3%), abdominal trauma (9%), surgical bleeding requiring re-operation (5.6%), intestinal ischemia (2.5%), and other indications (5.1%) (Fig. 2).

### Fascial closure (study endpoint)

The DFC rate was 57.5% (362 of 630 patients) in the intention-to-treat analysis and 71% (362 of 510 patients) in the per-protocol analysis, which excluded patients who had died during OAT (Table 2). There were significant differences in DFC rates (58–100%) between participating hospitals (*p* < 0.001), which, however, did not depend on OAT indications (Table 3).

**Table 2:** Fascial closure and univariate analysis of influencing factors

	Percentage of patients	Number of patients	*p* value
Complete fascial closure (per-protocol analysis)	71	(362/510)	
Complete fascial closure (intention-to-treat analysis)	57.5	(362/630)	
Range of fascial closure rates across hospital departments	58–100	(80/138–21/21)	< 0.001
Fascial closure rates according to OA indication			0.215
Trauma	80.9	(38/47)	
Peritonitis	66.2	(157/237)	
Abdominal compartment syndrome	76.8	(73/95)	
Burst abdomen	69	(40/58)	
Surgical bleeding	72.7	(24/33)	
Intestinal ischemia	90	(09/10)	
Other	70	(21/30)	
Polytrauma (yes/no)	87.5 vs. 69,9	(28/32 vs. 334/478)	0.033
Intra-abdominal contamination (yes/no)	66.2 vs. 75.1	(157/237 vs. 205/273)	0.028
Björck’s classification at the initiation of OAT			< 0.001
Grade IA	84.2	(80/95)	
Grade IB	90.4	(66/73)	
Grade IIA	75.6	(31/41)	
Grade IIB	66.4	(75/113)	
Grade III	55.6	(5/9)	
Grade IV	50	(5/10)	
Björck’s classification at the completion of OAT			< 0.001
Grade IA	87.6	(127/145)	
Grade IB	93.3	(14/15)	
Grade IIA	69.8	(81/116)	
Grade IIB	77.8	(7/9)	
Grade III	30	(3/10)	
Grade IV	60.4	(9/14)	
Duration of OAT			< 0.001
Short (1–2 reoperations + OAT < 1 week)	77	(194/252)	
Medium (3–6 reoperations + OAT 7–21 days)	81	(77/95)	
Long (7 or more reoperations + OAT > 21 days)	55.9	(90/161)	
VPL (yes/no)	79.1 vs. 57.5	(250/316 vs. 111/193)	< 0.001
NPWT (yes/no)	78.6 vs. 51.4	(287/365 vs. 74/144)	< 0.001
DCT (no/yes)	84.8 vs. 60.8	(184/217 vs. 175/288)	< 0.001
OAT with VPL + NPWT + DCT	85.8 vs. 61.1	(175/204 vs. 187/306)	< 0.001

*OAT* open abdomen treatment, *VPL* visceral protective layer, *NPWT* negative-pressure wound therapy, *DCT* dynamic closure techniques

**Table 3:** Results of the multivariate logistic regression analysis

	Regression coefficient (B)	Standard error	Wald	*df*	Sig	Exp(B)	95% CI for EXP(B)	
							Lower limit	Upper limit
All patients (*N* = 510)								
Intra-abdominal contamination	0.462	0.212	4.746	1	0.029	0.63	0.415	0.955
Surgical procedures before OAT initiation	0.302	0.106	8.022	1	0.005	0.74	0.6	0.911
NPWT	− 0.915	0.24	14.561	1	< 0.001	2.496	1.56	3.993
DCT	− 0.988	0.252	15.437	1	< 0.001	2.687	1.641	4.4
Only patients with peritonitis (*n* = 237)								
Surgical procedures before OAT initiation	0.434	0.162	7.161	1	0.007	0.648	0.471	0.89
NPWT	− 1.311	0.351	13.932	1	< 0.001	3.71	1.864	7.384
DCT	− 0.819	0.366	5.005	1	0.025	2.269	1.107	4.65
Only patients with ACS (*n* = 129)								
NPWT	− 2.293	0.64	12.831	1	< 0.001	9.9	2.824	34.706

*OAT* open abdomen treatment, *CI* confidence interval, *NPWT* negative-pressure wound therapy, *DCT* dynamic closure techniques, *ACS* abdominal compartment syndrome

There were 42 patients with a mesh at the end of OAT. 17 (40.5%) as a prophylactic mesh with a definitive fascial closure, 25 (59.5%) without a definitive fascial closure as a fascial bridging technique.

### The role of fascial closure

Successful DFC did not significantly reduce hospital length of stay (LOS) after OAT completion but was associated with a significant decrease in mortality from 23% (34 of 148 patients) to 14.1% (51 of 311 patients) (*p* = 0.015). In addition, the group of patients with DFC showed a significantly lower incidence of enteroatmospheric fistula (5.5% vs. 17.6%) (p < 0.001).

### Treatment course data for the entire patient population

#### Treatment before open abdomen treatment

A detailed overview of treatment characteristics is provided in Table 1.

#### Open abdomen treatment

In only 30.3% of the cases was OAT performed according to an established standard treatment protocol. In all other cases, the individual surgeon responsible for the patient decided how to manage the patient. The mean duration of OAT was 16.1 ± 24.9 days. During this period, a mean number of 3.9 ± 3.7 procedures were performed.

**Fig. 3:**
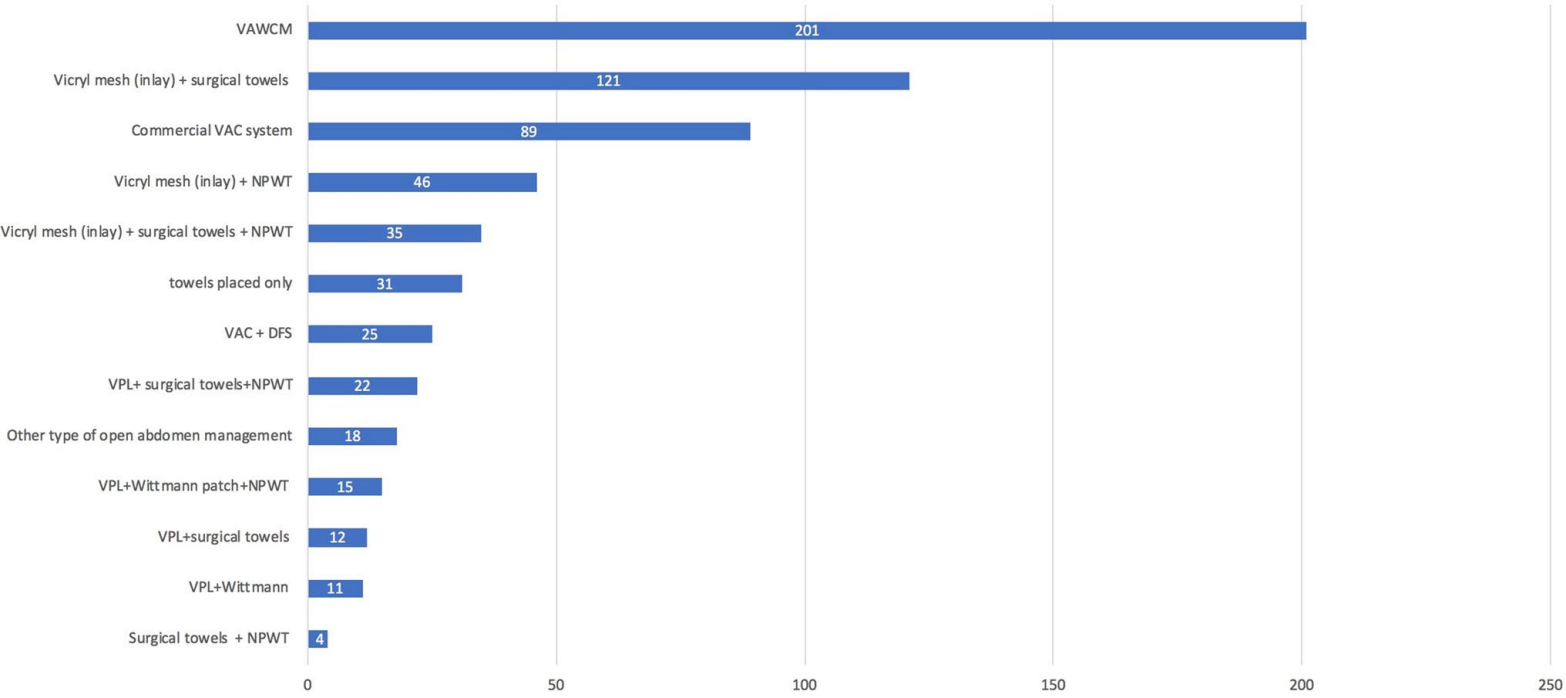
Open abdomen techniques (number of patients). *VAWCM* vacuum-assisted wound closure and mesh-mediated fascial traction, *VAC* vacuum-assisted closure, *NPWT* negative-pressure wound therapy, *VPL* visceral protective layer, *DFS* dynamic fascial sutures

**Fig. 4:**
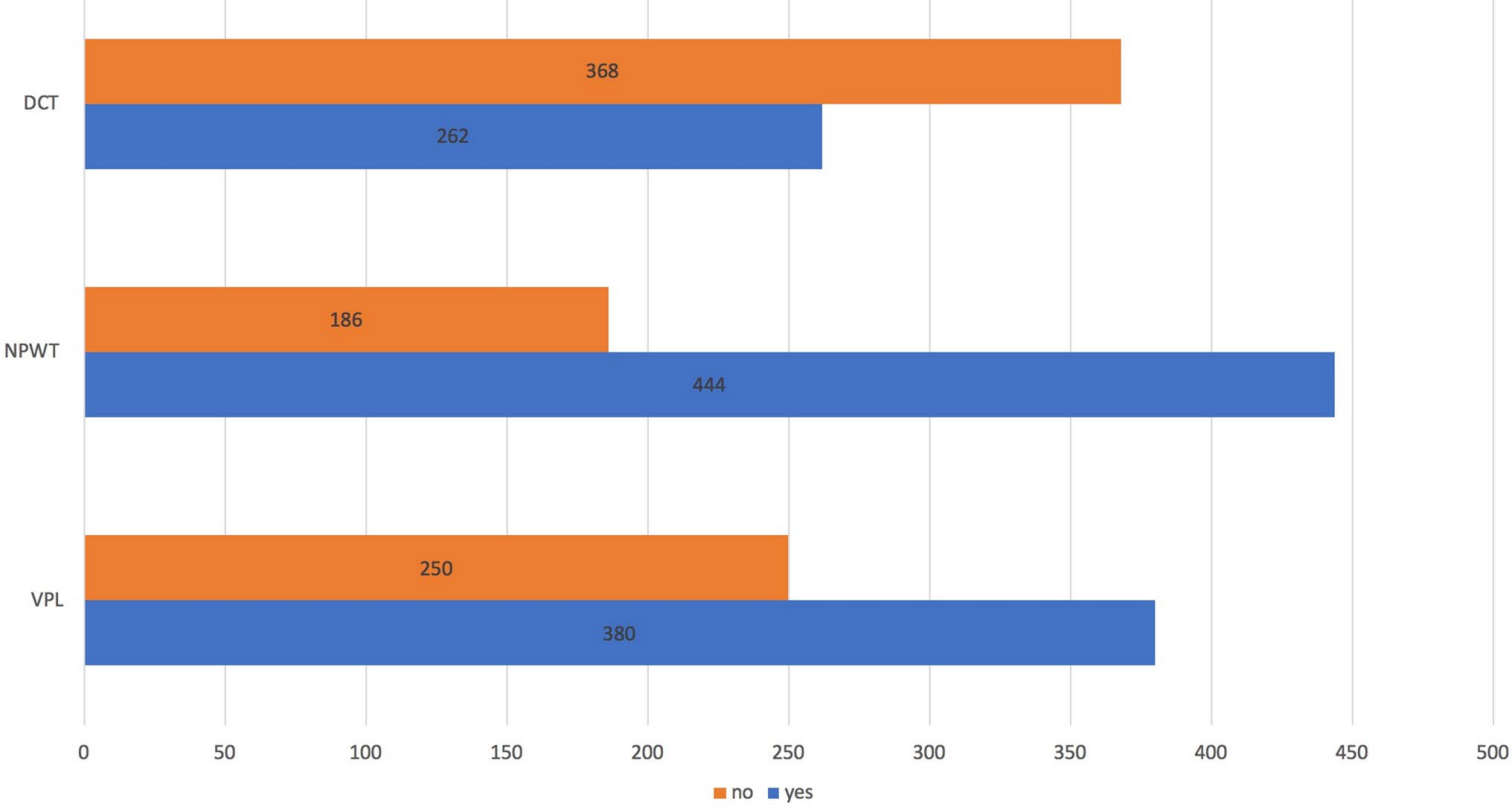
Open abdomen treatment elements (number of patients). *DCT* dynamic closure techniques, *NPWT* negative-pressure wound therapy, *VPL* visceral protective layer

Thirteen different open abdomen techniques were used in the management of the patient population studied here (Fig. 3). They are investigated according to the three predefined elements of treatment groups (Fig. 4).

#### Factors influencing DFC

A univariate analysis showed that polytrauma was associated with a significantly higher fascial closure rate than other indications for OAT (87.5% versus 69.9%, *p* = 0.033) whereas the presence of intra-abdominal contamination resulted in a significantly lower fascial closure rate than the absence of intra-abdominal contamination (66.2% versus 75.1%, *p* = 0.028). Likewise, higher-grading of the OA, based on Björck’s classification system, both at the initiation and at the completion of OAT, showed significantly lower fascial closure rates. Longer duration of OAT adversely affected fascial closure (Table 2). All three treatment elements (NPWT, DCT, VPL) were positively correlated with achieving fascial closure (Table 2). In particular, a combination of NPWT, DCT and VPL achieved the best overall results with the highest fascial closure rate compared to all other techniques not using this combination (85.8% vs. 61.1) (Table 2).

74.3% of the vacuum therapies were carried out using commercial systems, 16.9% using wall suction and 1.1% using other systems. There was no difference in the rate of fascial closure between the various manufacturers of the commercial systems. However, there was a significant difference between the commercial and improvised vacuum systems with wall suction (82.8% vs. 58.1%, *p* < 0.001).

The results of the multivariable logistic regression are shown in Table 3. In the last step of the regression, the use of NPWT and the use of DCT were independently correlated with the study endpoint DFC (Table 3). Intra-abdominal contamination and the number of surgical procedures before OAT initiation were negatively correlated with DFC (Table 3).

In a subgroup analysis, similar results were obtained for patients with peritonitis. NPWT and DCT were again associated with a higher probability of fascial closure, whereas the number of surgical procedures before OAT initiation was negatively correlated with DFC (Table 3).

In an analysis of the group of patients with ACS, which was the second largest patient group, only the use of NPWT was found to be significantly associated with successful DFC (Table 3).

## Discussion

The present study implements a systematic analysis of a multi-centric patient cohort with OAT concerning successful DFC as a key objective to achieve low mortality and morbidity. The main findings of this study emphasize the importance of a structured treatment plan and technical aspects to achieve high DFC rates.

Our data emphasize the role of DFC in the course of disease during the hospital stay and show that successful DFC is associated with lower mortality. A similar relationship was reported by von Websky et al. in a single-center study that, unlike the present study, analyzed overall mortality over the entire course of treatment [10]. Review studies comparing different techniques often demonstrate a positive correlation between DFC and survival [9, 25]. Moreover, this study confirms the results reported by Cristaudo et al. that showed that DFC also reduced the incidence of EAF [9, 25].

The composition of the patient population and the indications for OA management reflect those commonly found in similar cohort of patients in Europe: the most frequently reported indication for OA was peritonitis, followed by ACS, and burst abdomen. Trauma accounted for only 9% [4, 10, 11, 17, 36, 37]. In this study, an in-hospital mortality rate of 19% was in line with the literature on OAT, where it varies between 10 and 45% [14, 23, 36–41]. The fact that 36% of the patients in this study met the applicable criteria for sepsis at the initiation of OA underlines the fact that most of these patients were critically ill [33, 34].

A novel methodological aspect in this investigation is the identification and analysis of the independent roles of individual technical and patient-related factors and the summation of several surgical strategies into “three key elements” in OAT.

Some of these factors were already examined in former studies in single factorial analyses. Especially the impact of NPWT during OAT was shown concerning several treatment aspects [42, 43]. Despite many advantages, NPWT alone may not be able to sufficiently prevent fascial lateralization during OAT especially in patients with peritonitis and patients requiring long treatment courses [44]. As a result, mean fascial closure rates of 60% are reported in per-protocol analyses and only approximately 30% in some patient populations [25, 45, 46].

In a large multi-centric study by Carlson, vacuum therapy alone was even inferior to a mixed patient cohort treated with other techniques with and without DCT with regard to DFC [37].

In 2017, Acosta et al. found in a review article including eleven observational studies that high fascial closure rates can be achieved with VAWCM even in elderly non-trauma patients, most of whom presented with peritonitis [16]. This method was first described in 2007 [39]. This result was confirmed by other authors like Cristaudo et al. and Atema et al. concerning different types of patient populations. They found that a combination of NPWT and DCT was superior to NPWT alone with regard to DFC [9]. In their clinical guidelines on the management of the abdominal wall in the context of the open or burst abdomen, the EHS too recommended the use of DCT and NPWT on the basis of a meta-analysis but reported that the level of evidence was low [27].

In this study, the highest fascial closure rates were obtained for the total patient population when OAT combined the three key treatment elements NPWT, VPL, and DCT.

This result applies to both the total patient population and the subgroup of patients with peritonitis. By contrast, NPWT alone is successful in patients with ACS. The different pathophysiologies are a likely explanation for this finding since NPWT is the treatment element to reduce bowel edema [42].

Intra-abdominal contamination and the number of surgical procedures before OAT were found to be the only factors that were negatively correlated with DFC. This result confirms the previous observation reported by Karhof et al. [4].

Moreover, patient-related factors were not significantly associated with DFC in the multivariable analysis. There was a difference in DFC rates between the different indications and we obtained the highest rates for trauma patients (more than 80%), as did earlier studies, but the difference was not statistically significant [11, 36, 47].

Our findings and recent results from the literature concerning the role of a visceral protective layer (VPL) in the prevention of fistulas suggest that an ideal approach to OAT consists of a combination of VPL, NPWT and DCT [30]. As a result, maximum synergistic effects can be achieved through a combination of fascial traction using DCT, edema reduction using NPWT, and the prevention of adhesions and fistula formation using VPL.

Taken together, available data support a fundamental role for technique-related factors that, independently of each other, have positive effects on DFC. Both DCT and NPWT were found to be independent factors that contribute to successful DFC.

The present study furthermore reveals the use of a wide diversity of techniques (13 different techniques) and a low level of treatment standardization (approximately 30% of the cases). The best results, however, have so far been reported in those studies that showed the highest level of in-hospital standardization [11, 17, 19, 36, 48]. A growing body of evidence from meta-analyses and registry-based studies that have been conducted in recent years, however, will probably lead to an increase in in-hospital standardization [9, 26, 29, 49, 50].

## Conclusions

The present study is the first systematic multi-center analysis of different factors that promote or prevent fascial closure after OAT. It found that the three principles DCT, NPWT and the use of a VPL resulted in the highest DFC rates. Two of the four factors that were found to be significantly associated with fascial closure are not patient-related but treatment-related factors. These factors can be influenced by the surgeon, i.e. NPWT and DCT. An integrative approach combining the above principles allows surgeons to actively and positively promote the course of OAT and reduce the risks and complications potentially associated with it.

### Limitations

Basically, there is a strong heterogeneity of the individual patient groups in the individual clinics. The classification into the treatment elements serves the purpose of improving comparability and analysis in this respect. In the absence of sufficiently large numbers of patients, a multivariate analysis has not yet been performed with a view to assessing the effects of a variety of factors on DFC as a study endpoint. For this reason, comparisons with other studies (single-center observational studies, meta-analyses, etc.) are subject to limitations from a methodological perspective.

## Electronic supplementary material

Below is the link to the electronic supplementary material.

Supplementary file1 (DOCX 15 KB)

Supplementary file2 (XLSX 33 KB)
