# Supplementary material for: Factors influencing the fascial closure rate after open abdomen treatment: Results from the European Hernia Society (EuraHS) Registry: Surgical technique matters
Source: Hernia. 2020 Nov 21;26(1):61–73. doi: 10.1007/s10029-020-02336-x (PMC8881440; doi:10.1007/s10029-020-02336-x)
Supplement: Supplementary file 1 — Supplementary file1 (DOCX 15 KB) [file 10029_2020_2336_MOESM1_ESM.docx]

**Supplement 2: List of participating hospitals and departments**

- Department of General, Visceral, Transplantation, Vascular and Pediatric Surgery, University Hospital of Würzburg, Germany
- Department of General, Visceral and Thoracic Surgery, German Armed Forces Central Hospital, Koblenz, Germany
- Department of General, Visceral, Thoracic and Vascular Surgery, University Hospital of Bonn, Germany
- Department of General and HPB Surgery and Liver Transplantation, Ghent University Hospital, Belgium
- Emergency Surgery Unit, Cisanello University Hospital, Pisa, Italy
- Department of Surgical Sciences, Section of Vascular Surgery, Uppsala University, SE 751 85 Uppsala, Sweden
- Department of General, Visceral and Oncological Surgery, Wilhelminenspital, Vienna, Austria
- Department of General Surgery, Sainte Anne Military Hospital, Toulon, France
- Department of Surgery, Regensburg University Hospital, Germany
- Department of Abdominal, Vascular and Transplant Surgery, Cologne-Merheim Medical Center, Witten/Herdecke University, Cologne, Germany
- Department of Surgery, Maria Middelares Hospital, Ghent, Belgium
- Department of General, Visceral and Thoracic Surgery, German Armed Forces Hospital of Ulm, Oberer Eselsberg, Ulm, Germany
